# Functional near-infrared spectroscopy characteristics in children with autism spectrum disorder under animated video modeling therapy

**DOI:** 10.3389/fneur.2025.1590185

**Published:** 2025-07-25

**Authors:** Chuanhua Zhu, Hongwei Li, Lina Zhang, Yonglu Liu, Yangyang Zhang, Binbin Huang, Wei Li

**Affiliations:** ^1^Department of Rehabilitation, Binzhou Medical University Hospital, Binzhou, China; ^2^School of Special Education and Rehabilitation, Department of Rehabilitation Medicine, Binzhou Medical University, Yantai, China

**Keywords:** autism spectrum disorder, animated video modeling, brain functional connectivity, visual stimulation, functional near-infrared spectroscopy

## Abstract

**Objective:**

To investigate the impact of animated video modeling (AVM) on the brain function of children with autism spectrum disorder (ASD) using functional near-infrared spectroscopy (fNIRS).

**Methods:**

Fifteen children with ASD and 15 matched typically developing (TD) controls were enrolled. fNIRS was used to obtain 8-min data in quiet and visually stimulated states, with the dorsolateral prefrontal cortex, medial prefrontal cortex (mPFC), and bilateral occipital lobe as regions of interest (ROIs). Based on the concentration of oxygenated hemoglobin (HbO_2_) over time, correlation coefficient analysis was performed to calculate functional connection strength, and the intergroup disparity was compared.

**Result:**

The ASD group showed significantly lower functional connection strength. Comparison of the ROI–ROI functional connectivity strength revealed significant differences in connectivity patterns of the right dorsolateral prefrontal lobe (RDLPFC), left dorsolateral prefrontal lobe (LDLPFC), mPFC, right occipital lobe (ROL), and left occipital lobe (LOL) with other brain regions. Extremely significant differences were found between the RDLPFC/RPMC, ROL/RPMC, LOL/RPMC, and LOL/LIPL. The functional connectivity strength of children with ASD was significantly higher during visual stimulation than during the quiet test. Comparison between the ROI–ROI functional connectivity strengths revealed significant differences in the connectivity strength of the RDLPFC/LOL, LDLPFC/LIPL, mPFC/RPMC, mPFC/LPMC, mPFC/LIPL, ROL/RPM, ROL. RIPL, ROL/LIPL, and LOL/LIPL. Extremely significant differences were observed between the ROL/RPMC and ROL/RIPL.

**Conclusion:**

Animated video modeling can improve visual perception and information processing in children with ASD, by strengthening the functional connectivity between the occipital and inferior parietal cortices.

## Introduction

Autism spectrum disorder (ASD) encompasses a group of neurodevelopmental disorders characterized by varying degrees of impairment in social interaction and communication, restricted interests, and repetitive behaviors (1). According to data statistics from the Autism Spectrum and Developmental Disorders Surveillance (ADDM) Network, the incidence of ASD is increasing annually, with this disease emerging as a significant global issue in pediatric neurodevelopmental disorders (2). Visual perception and information processing abnormalities are common symptoms in children with ASD, significantly affecting their daily functioning and learning abilities and impeding their interaction with the environment and peers, resulting in varying degrees of social interaction and communication disorders. Yeung et al. (3) discovered that children with ASD frequently have difficulty recognizing and interpreting facial expressions, which can impede social interactions and the development of relationships. Similarly, Dakin et al. (4) reported that numerous children with ASD display abnormal patterns of visual attention, such as fixating on specific details rather than the entire scene, and that this tendency can interfere with their ability to perceive and interpret social cues. Furthermore, Posar et al. (5) noted that the integration of visual information from different sources can be problematic for children with ASD, affecting their ability to understand complex visual scenes and interactions. Therefore, understanding and addressing these visual processing deficits is crucial for developing effective interventions that can improve social interaction, communication impairments, and cognitive outcomes among children with ASD (6, 7). Owing to patients’ relatively strong dependence in responding to visual cues (8), an increasing number of educational programs are incorporating visual teaching and learning strategies (9, 10).

Animated video modeling (AVM) is a visual aid that can effectively impact the social skills of children with ASD (11). Visual dependence has been indicated to strongly influence several individuals with ASD. Children with ASD often show a keen interest in videos, acquiring significant knowledge from them (12, 13). Numerous researchers have observed a preference for visual stimuli, particularly those presented on electronic screens. This has led to speculation that this preference can be exploited to further enhance various skills (14). Some researchers have further proposed that the specific characteristics of electronic screen media make them effective tools for delivering information to individuals with ASD. The limited viewing area helps narrow the participants’ attention frame, enabling them to focus on relevant stimuli and disregard irrelevant ones (15). Many educators use this preference for electronic screen media in teaching, with video modeling being one of the most effective applications. This intervention method can help children with ASD develop various skills. This process involves recording the modeling of target behaviors and skills into videos for students to repeatedly watch and imitate, thus facilitating the acquisition of these behaviors and skills (13, 16, 17). However, the functional mechanisms underlying the action of AVM in improving social and communication skills in children with ASD remain unclear.

Functional near-infrared spectroscopy (fNIRS) is an ideal neuroimaging technique for investigating brain function in children. Owing to its advantages of affordability, portability, minimal noise interference, and high tolerance to head movements, this technique has gained popularity among many researchers and has been extensively used in the study of brain functional connectivity and activation in individuals with ASD (18–21).

The present study collected different state data of 3–7-year-old children diagnosed with ASD and matched typically developing (TD) children using fNIRS to investigate the AVM impact on brain function in children with ASD by comparing the magnitude of functional strength between the two groups.

## Materials and methods

### Participants

The ASD group comprised children diagnosed with ASD at the Binzhou Medical University Hospital between October 2023 and April 2024. The inclusion criteria were as follows: Patients who met the criteria of the Diagnostic and Statistical Manual of Mental Disorders, 5th Edition (DSM-5); aged between 3 and 7 years; right-handedness; and no discomfort experienced for nearly 1 month. The exclusion criteria were as follows: Other neurodevelopmental disorders, inability to complete fNIRS, and a history of transcranial magnetic stimulation or other neuroregulatory treatments. A total of 15 children with ASD met the inclusion criteria. Concurrently, 15 TD children enrolled in the child rehabilitation department were included in the TD group.

In addition, all of the participants’ parents were requested to report their child’s behavior using the Autism Behavior Checklist (ABC), and a parental reporting questionnaire, was used to confirm the clinical diagnosis of ASD. Fifty-seven items describing typical behaviors in different developmental areas of children with ASD were included and divided into five subscales (sensory, relating, body and object use, language, and social and self-help skills). Each item was scored on a scale from 1 to 4, depending on its importance for the diagnosis of ASD. The subscale scores were determined by summing the values of the endorsed items for each area, which were then summed to represent the total ABC score. Children with a total score of 68 points may have a high risk of ASD.

The study was conducted following approval by the Medical Ethics Committee of Binzhou Medical University Hospital (approval number 2024-KYLL-141). All methods were performed in accordance with the relevant guidelines and regulations, including the Declaration of Helsinki and institutional ethical standards. Written informed consent was obtained from all participants and/or their legal guardians for both study participation and publication of identifying information/images in an online open-access publication. No patient names or other identifying details are included in this manuscript. All images used in this study are fully anonymized, adhering to ethical standards to protect participant privacy.

### Data acquisition

**Figure 1:**
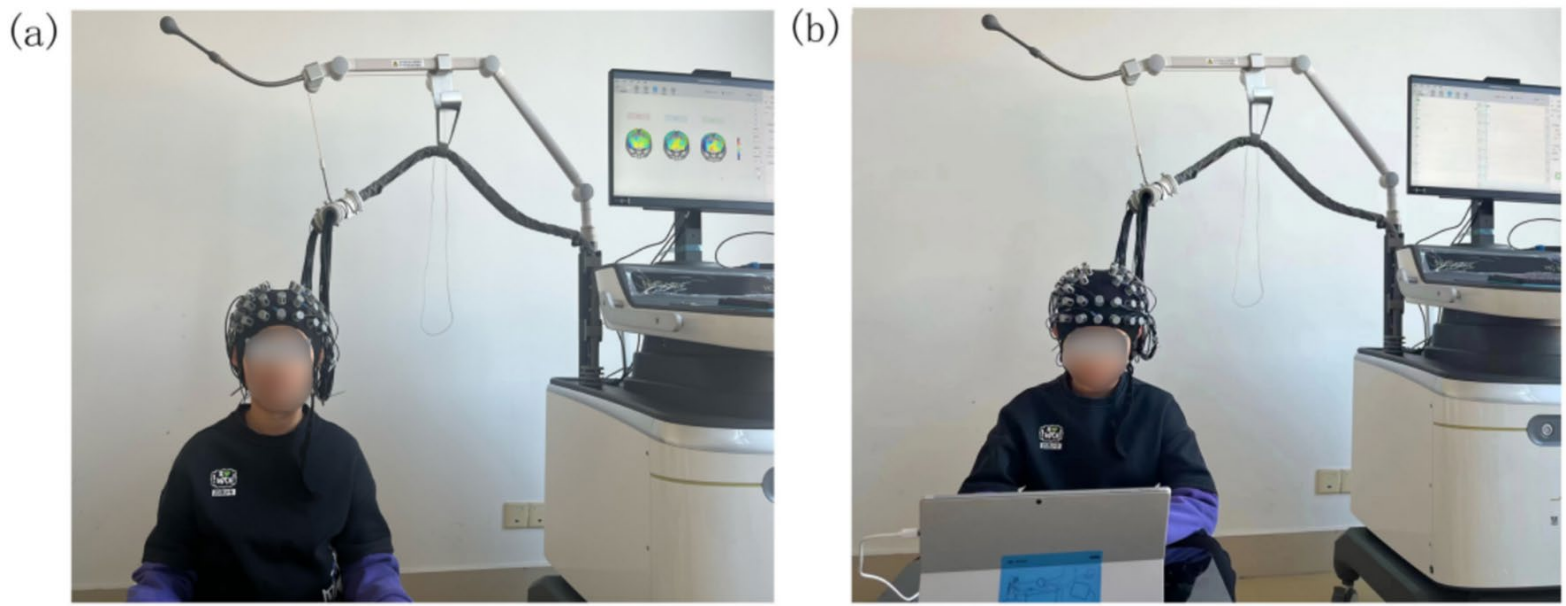
Schematic diagram of experimental data acquisition. **(a)** Photo obtained from a participant during data collection in the quiet state. **(b)** Photo obtained from a participant during data collection in the visual stimulation state.

NirScan-9000ET (Danyang Huichuang Medical Equipment Co., Ltd., China) was applied to continuously measure and record brain oxyhemoglobin (HbO_2_) and deoxyhemoglobin (Hb) concentration changes in participants in a quiet state and during visual stimulation from the AVM. The sampling frequency was 11 Hz. The device comprised 24 transmitting and 24 receiving probes with 63 effective channels; the channel coordinates were based on the standard international 10–20 electrode system. During data collection, eight resting-state data points were collected while participants either sat alone or were held by a guardian (Figure 1).

**Figure 2:**
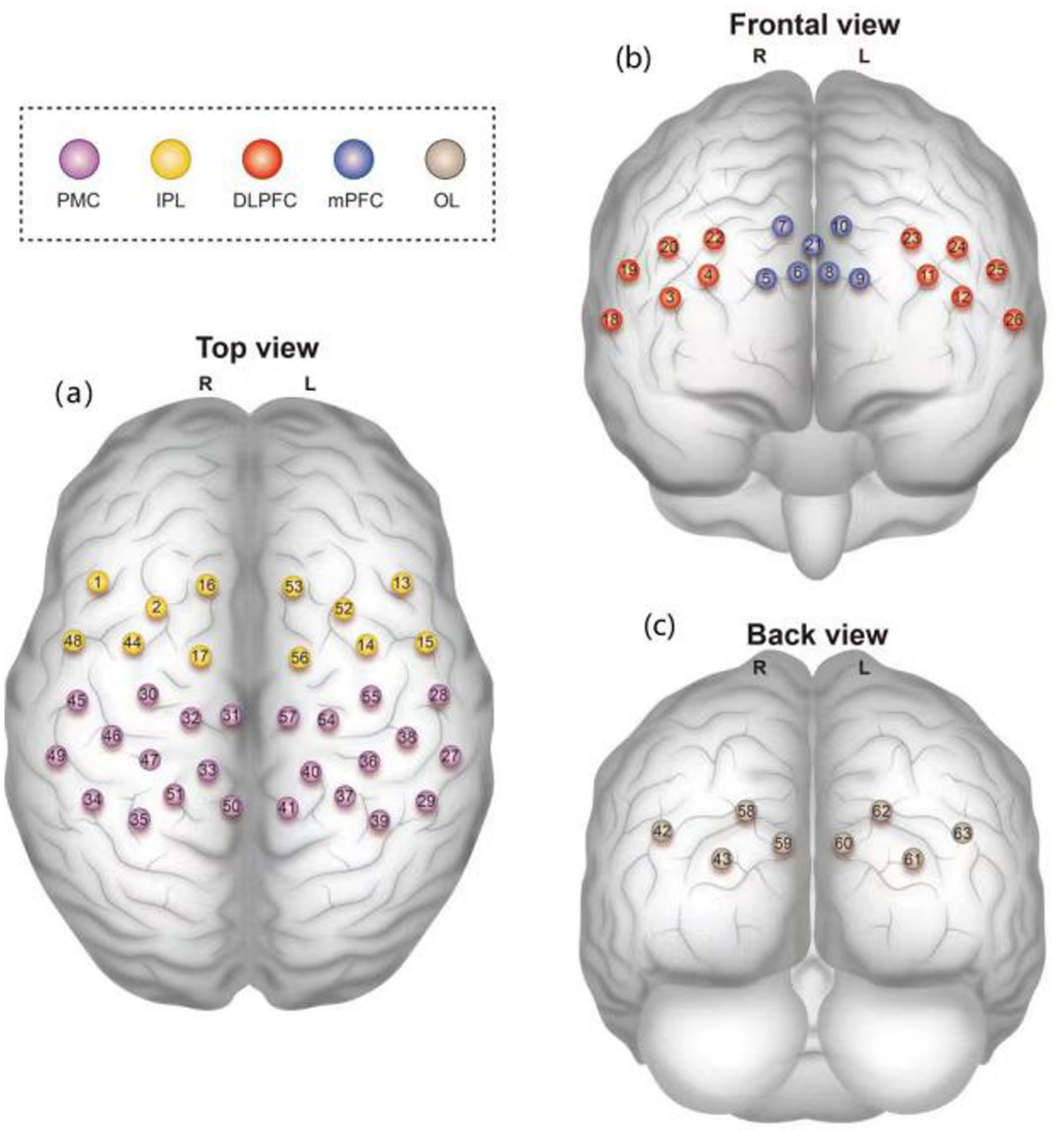
**(a)** Top view, **(b)** Frontal view, and **(c)** Back view of the brain regions covered by 63 fNIRS channels. Color-coded regions: dorsolateral prefrontal cortex (DLPFC, red), medial prefrontal cortex (mPFC, blue), premotor cortex (PMC, purple), inferior parietal lobe (IPL, yellow), and occipital lobe (OL, gray).

In this study, the brain was divided into the dorsolateral prefrontal cortex (DLPFC), medial prefrontal cortex (mPFC), bilateral premotor cortex (PMC), inferior parietal lobe (IPL), and bilateral occipital lobe (OL), of which the bilateral DLPFC, mPFC, and OL were used as regions of interest (ROIs) (Figure 2). The HbO_2_ concentration change signal was selected as the primary data collected for the test because of its high sensitivity to cortical blood flow changes and superior signal-to-noise ratio (11).

### Animation modeling video

Videos that met the following content criteria were selected: (a) engaging a partner in an interactive play, (b) initiate and sustain conversations with a partner, and (c) start and maintain enjoyable activities with a partner. The videos showcased peers exhibiting four fundamental behaviors for each task, as follows: (1) interaction and engagement by maintaining eye contact while speaking; (2) coherence through nonverbal communication, such as direct facial expressions (e.g. smiling or frowning), to convey interest; (3) peer-to-peer conversation taking turns engaging in dialogue; and (4) politeness and etiquette by offering additional comments or asking questions related to the interlocutor’s statements.

### Data analysis

NirSpark software was used for data preprocessing and analysis. During preprocessing, a quality control module was used to detect motion artifacts and filter unqualified data. The spline interpolation method was used to correct movement artifacts in the channel. Physiological noise was eliminated using a low-pass filter at approximately 0.009–0.008 Hz (22–24), while the filtered optical density data were converted into HbO_2_ concentration changes according to the modified Beer–Lambert law.

Functional connectivity analysis: Data from each time point were extracted for each participant, and a statistical analysis of the HbO_2_ concentration in each brain region over the time series was conducted. The Pearson correlation method was applied to calculate the correlation between each channel and brain region, with the correlation coefficient “r” of HbO_2_ valued by Fisher-Z conversion as a measure of the functional connectivity strength for each corresponding channel. Moreover, comparisons were made regarding the functional connectivity strength between ROI-ROIs.

### Statistical methods

SPSS (version 25.0) software was used for statistical analysis. Data with normal distribution, such as age and functional connectivity strength, are described as the x- ± s. Independent sample t-tests were used to compare two groups, whereas paired sample t-tests were used to compare different states in the same group. The test level was set at *α* = 0.05 for bilateral tests.

## Results

### Demographic and behavioral questionnaires

No significant differences in age and gender were observed between the ASD and TD groups, (*p* > 0.05), while the childhood autism rating scale (CARS) was significantly lower in the TD groups, compared with ASD group (**Table** **1**). The subscales and total ABC scores of all children with ASD included in the data analysis were significantly higher than those of TD children (*p* < 0.05) (Table 1).

**Table 1:** Demographic and clinical characteristics of children with ASD and TD children.

Variable	ASD	TD	*p*-value
Age (years), mean ± SD	4.47 ± 0.92	4.73 ± 0.88	0.797
Gender			1.0
Male	3 (20.0%)	3 (20.0%)	
Female	12 (80.0%)	12 (80.0%)	
CARS, mean ± SD	34.07 ± 2.25	15.33 ± 0.49	<0.001
ABC score, mean ± SD			
Sensory	19.9 ± 3.3	1.9 ± 2.6	<0.001
Relating	22.4 ± 7.6	1.7 ± 2.6	<0.001
Body and object use	21.2 ± 5.7	0.8 ± 1.0	0.002
Language	13.6 ± 4.9	1.2 ± 1.0	0.003
Social and self-help skills	13.5 ± 3.4	2.9 ± 1.2	0.003
Total score	89.9 ± 18.0	8.3 ± 3.8	<0.001

ASD: autism spectrum disorder, TD: typically developing.

### Functional connectivity in children with ASD and TD children

**Figure 3:**
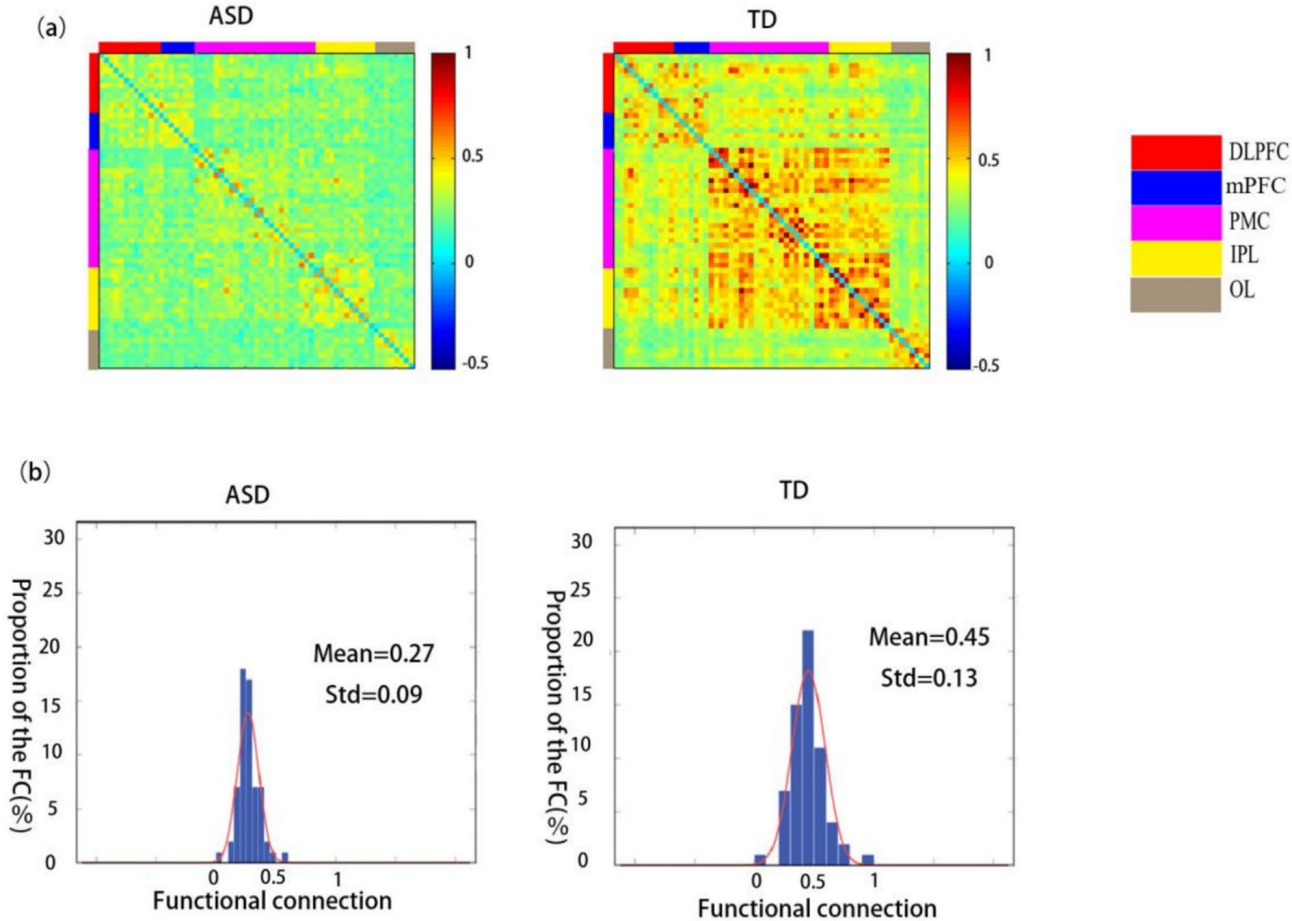
Spatial patterns of functional connectivity in the ASD and TD groups. **(a)** Functional connectivity maps for the ASD and TD groups. **(b)** Histograms of the functional connectivity distribution in the ASD and TD groups. TD: typically developing, ASD: autism spectrum disorder.

A comparison between the functional connectivity maps of the ASD and TD groups revealed that the average functional connectivity strength was lower in the ASD group (0.27 ± 0.09) than in the TD group (0.45 ± 0.13) (Figures 3a,b).

Comparison of the ROI–ROI functional connectivity strength between children in the ASD and TD groups revealed statistically significant differences in the connectivity patterns of the right dorsolateral prefrontal lobe (RDLPFC), left dorsolateral prefrontal lobe (LDLPFC), medial prefrontal cortex (mPFC), right occipital lobe (ROL), and left occipital lobe (LOL) with other brain regions (*p* < 0.05). Extremely significant differences were found between the RDLPFC and RPMC, ROL and RPMC, LOL and RPMC, as well as between the LOL and LIPL (*p* < 0.01) (Table 2).

**Table 2:** Comparison of ROI–ROI rsFC intensity between the TD and ASD groups.

ROIs	TD	ASD	*p*-value	*t*
RDLPFC ~ LDLPFC	0.41 ± 0.15	0.30 ± 0.1	0.030^*^	2.312
RDLPFC ~ RPMC	0.40 ± 0.15	0.25 ± 0.08	0.003^**^	3.246
RDLPFC ~ LPMC	0.36 ± 0.16	0.24 ± 0.07	0.015^*^	2.677
RDLPFC ~ RIPL	0.42 ± 0.17	0.29 ± 0.11	0.015^*^	2.604
RDLPFC ~ LIPL	0.35 ± 0.15	0.25 ± 0.1	0.043^*^	2.122
RDLPFC ~ LOL	0.26 ± 0.09	0.2 ± 0.07	0.042^*^	2.133
LDLPFC ~ RPMC	0.36 ± 0.19	0.24 ± 0.08	0.029^*^	2.306
LDLPFC ~ LPMC	0.36 ± 0.18	0.26 ± 0.07	0.045^*^	2.099
LDLPFC ~ RIPL	0.36 ± 0.19	0.25 ± 0.08	0.041^*^	2.143
LDLPFC ~ LIPL	0.37 ± 0.18	0.26 ± 0.08	0.047^*^	2.082
LDLPFC ~ mPFC	0.43 ± 0.14	0.32 ± 0.09	0.016^*^	2.557
mPFC ~ LPMC	0.36 ± 0.07	0.24 ± 0.07	0.016^*^	2.554
mPFC ~ RIPL	0.37 ± 0.17	0.24 ± 0.09	0.015^*^	2.600
mPFC ~ LIPL	0.37 ± 0.24	0.24 ± 0.11	0.017^*^	2.534
ROL ~ RPMC	0.31 ± 0.11	0.20 ± 0.05	0.003^**^	3.407
ROL ~ LOL	0.46 ± 0.25	0.28 ± 0.16	0.030^*^	2.303
LOL ~ RDLPFC	0.26 ± 0.09	0.20 ± 0.07	0.042^*^	2.133
LOL ~ RPMC	0.32 ± 0.13	0.21 ± 0.08	0.007^**^	2.959
LOL ~ RIPL	0.34 ± 0.14	0.25 ± 0.08	0.028^*^	2.325
LOL ~ LIPL	0.33 ± 0.12	0.23 ± 0.07	0.006^**^	2.946
LOL ~ mPFC	0.27 ± 0.08	0.20 ± 0.08	0.019^*^	2.490

TD: typically developing, ASD: autism spectrum disorder, ROI: region of interest, RDLPFC: right dorsolateral prefrontal lobe, LDPFC: left dorsolateral prefrontal lobe, mPFC: medial prefrontal cortex, RPMC: right premotor cortex, LPMC: left premotor cortex, RIPL: right inferior parietal lobe, LIPL: left inferior parietal lobe, ROL: right occipital lobe, LOL: left occipital lobe. *p < 0.05, significant difference; **p < 0.01, large significant difference.

### Functional connectivity strength in children with ASD during quiet and visual stimulation

**Figure 4:**
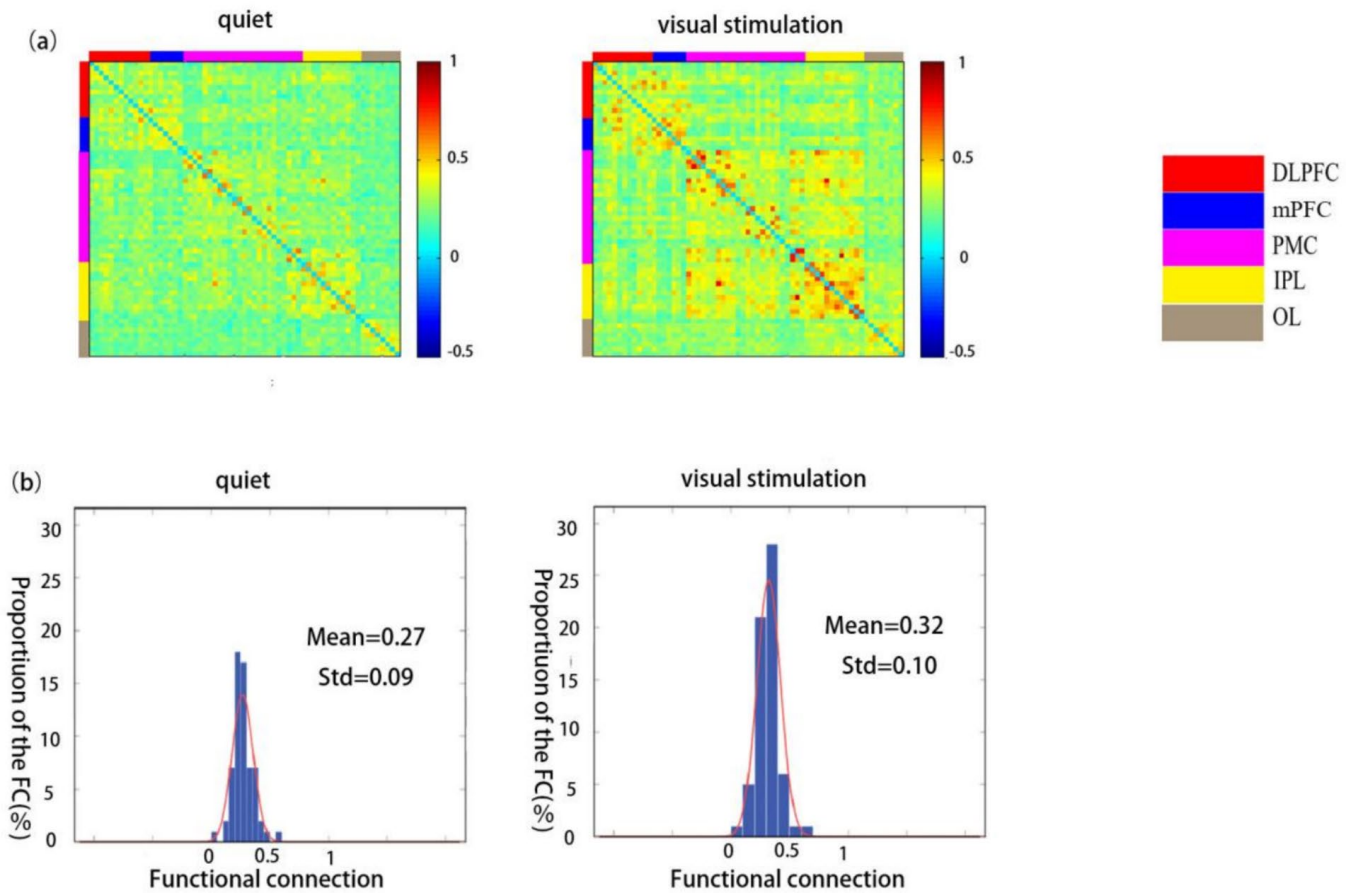
Spatial patterns of functional connectivity in the ASD group in the quiet and visual stimulation states. **(a)** Functional connectivity maps for the ASD group. **(b)** Histograms of the functional connectivity distribution. TD: typically developing, ASD: autism spectrum disorder.

The functional connectivity strength of children in the ASD group was significantly higher during the visual stimulation state (0.32 ± 0.10) than during the quiet state (0.27 ± 0.09) (Figures 4a,b).

A comparison between the ROI-ROI functional connectivity strength revealed statistically significant differences in the connectivity strength of the RDLPFC and LOL, LDLPFC and LIPL, mPFC and RPMC, mPFC and LPMC, mPFC and LIPL, ROL and RPM, ROL and RIPL, ROL and LIPL, as well as between the LOL and LIPL (*p* < 0.05). Further, extremely significant differences were observed between ROL and RPMC and ROL and RIPL (*p* < 0.01) (Table 3).

**Table 3:** Comparison of ROI–ROI functional connectivity strength between the quiet and visual stimulation states in children with ASD.

ROI	Quiet	Visual stimulation	*p*-value	*t*
RDLPFC ~ LOL	0.20 ± 0.07	0.24 ± 0.09	0.022^*^	−2.571
LDLPFC ~ LIPL	0.26 ± 0.08	0.31 ± 0.10	0.042^*^	−2.234
mPFC ~ RPMC	0.22 ± 0.08	0.26 ± 0.09	0.046^*^	−2.185
mPFC ~ LPMC	0.24 ± 0.07	0.27 ± 0.07	0.013^*^	−2.853
mPFC ~ LIPL	0.24 ± 0.11	0.29 ± 0.10	0.025^*^	−2.501
ROL ~ RPMC	0.20 ± 0.05	0.27 ± 0.07	0.001^**^	−4.452
ROL ~ RIPL	0.24 ± 0.07	0.33 ± 0.08	0.009^**^	−3.009
ROL ~ LIPL	0.22 ± 0.07	0.28 ± 0.11	0.024^*^	−2.524
LOL ~ LIPL	0.23 ± 0.07	0.31 ± 0.14	0.041^*^	−2.257

ASD: autism spectrum disorder, ROI: region of interest, RDLPFC: right dorsolateral prefrontal lobe, LDPFC: left dorsolateral prefrontal lobe, mPFC: medial prefrontal cortex, RPMC: right premotor cortex. *p < 0.05, significantly different, *p < 0.01, extremely significantly different.

### Functional connectivity strength in TD children during quiet and visual stimulation states

**Figure 5:**
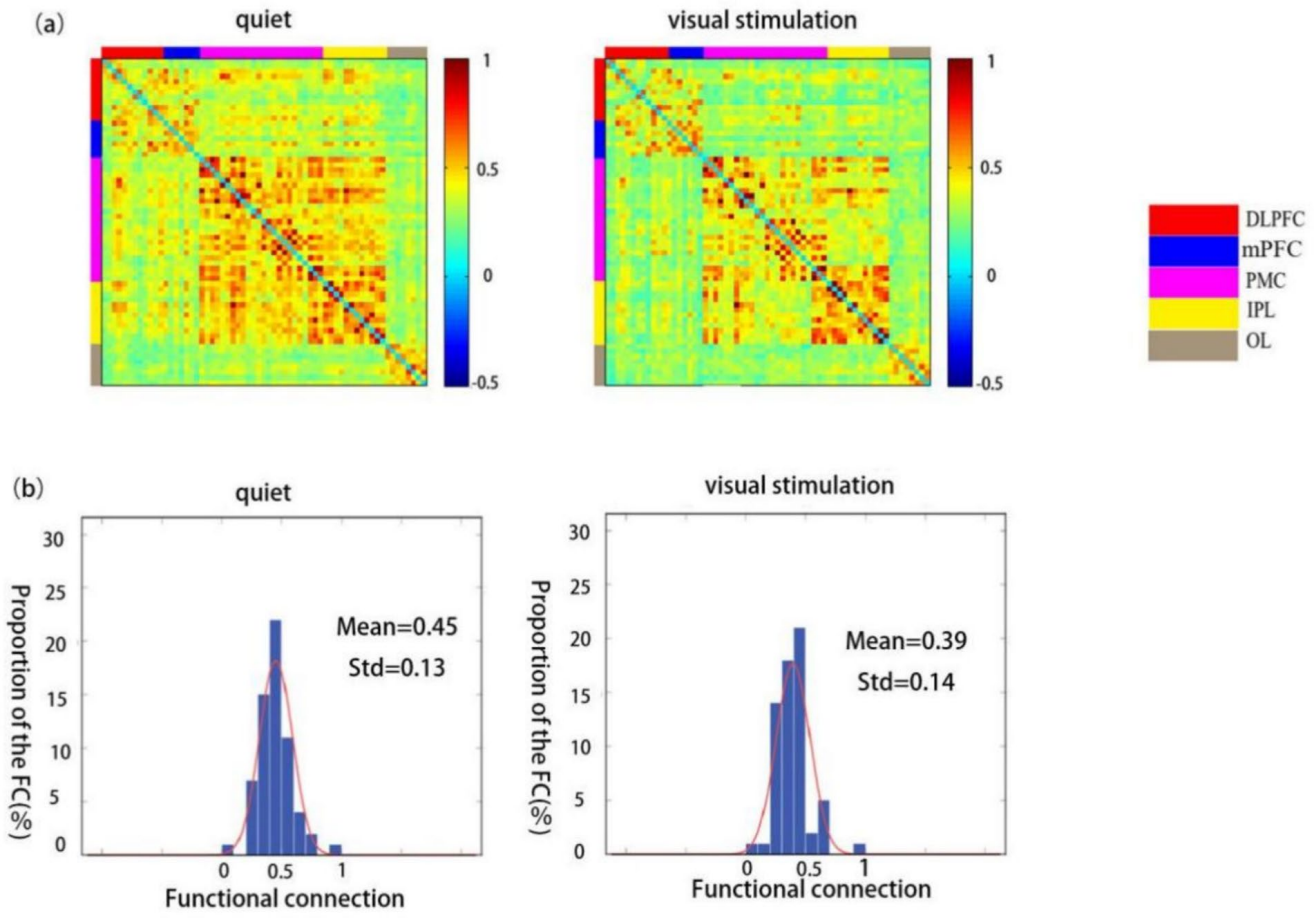
Spatial patterns of functional connectivity in the ASD group during quiet and visual stimulation state. **(a)** Functional connectivity maps of the ASD group. **(b)** Histograms of the functional connectivity distribution. TD: typically developing, ASD: autism spectrum disorder.

The functional connectivity strength of children in the TD group was significantly lower during visual stimulation (0.39 ± 0.14) than during the quiet state (0.45 ± 0.13) (Figures 5a,b).

The comparison between the ROI–ROI functional connectivity strength revealed statistically significant differences in the connectivity strength of the RDLPFC and RPMC, RDLPFC and RIPL, LDLPFC and ROL, LDLPFC and LIPL, mPFC and RPMC, mPFC and RIPL, and mPFC and LIPL regions (*p* < 0.05). Extremely significant differences were observed between the RDLPFC and RIPL (*p* < 0.01) (Table 4).

**Table 4:** Comparison of functional connectivity strength of ROI–ROI between the quiet and visual stimulation states in TD children.

ROIs	Quiet	Visual stimulation	*p*-value	*t*
RDLPFC ~ RPMC	0.4 ± 0.15	0.31 ± 0.06	0.028^*^	2.454
RDLPFC ~ RIPL	0.42 ± 0.17	0.32 ± 0.12	0.003^**^	3.642
LDLPFC ~ ROL	0.27 ± 0.07	0.22 ± 0.07	0.044^*^	2.217
LDLPFC ~ LIPL	0.26 ± 0.13	0.16 ± 0.14	0.044^*^	2.101
mPFC ~ RPMC	0.37 ± 0.17	0.29 ± 0.08	0.037^*^	2.308
mPFC ~ RIPL	0.37 ± 0.17	0.29 ± 0.11	0.048^*^	2.161
mPFC ~ LIPL	0.36 ± 0.17	0.30 ± 0.13	0.030^*^	2.413

TD: typically developing, ROI: region of interest, RDLPFC: right dorsolateral prefrontal lobe, LDPFC: left dorsolateral prefrontal lobe, mPFC: medial prefrontal cortex, RPMC: right premotor cortex, RIPL: right inferior parietal lobe, LIPL: left inferior parietal lobe, ROL: right occipital lobe. *p < 0.05, significant difference; **p < 0.01, large significant difference.

## Discussion

In recent years, an increasing number of studies have shown that ASD is associated with various brain neural network dysfunctions. Thus, the analysis of brain function connectivity in a resting state based on ROIs has emerged as a prominent topic in ASD research. This study used fNIRS to investigate brain functional connectivity strength in children with ASD and TD aged 3–7 years during the quiet test and while undergoing visual stimulation using AVM. These findings revealed that brain functional connectivity strength was lower in children with ASD than in TD children, indicating its potential as an effective neurobiological marker for the early detection of ASD.

Our ROI functional connectivity analysis revealed that connectivity between the DLPFC and other brain regions, as well as between the mPFC and other brain regions, was diminished in the ASD group. These findings align with previous research outcomes. For example, Rudie et al. (25) used resting-state fMRI to study children and adolescents with ASD and healthy controls and found that the functional connectivity between the prefrontal lobe and other brain regions in children with ASD was reduced. Zhu et al. (26) used fNIRS to show that the functional connection between the prefrontal lobe and other brain areas in children with ASD was weaker than that in healthy children in a common attention state. Mamashli et al. (27) also observed that the functional connections between the bilateral inferior frontal gyrus and temporal lobe were weaker in children with ASD than in healthy controls. Although the age of the participants and the research methods in this study were not completely consistent with those in the previously mentioned studies, we nevertheless found that children with ASD had reduced functional connectivity between ROIs. The decreases in functional connection strength observed in this study were all associated with the DLPFC and mPFC. The PFC plays a key role in higher cognition and working memory. This brain region has extensive neural fiber cross-projection connections and functional connections with other cerebral cortices and subcortical structures, making it dominant in the generation of related behaviors (28). Thus, we speculated that a decrease in the strength of functional connections between the PFC and other brain regions is the main cause of the decreased efficiency of the functional connection network in individuals with ASD.

Recently, AVM has been confirmed to be an effective teaching method for improving behavior in children with ASD. Video demonstration is commonly used to train children with ASD in various skills, mainly social behavior, functional life skills, cognitive behavior training, and problem behavior correction (29–31). Gaffrey and Samson also noted that individuals with ASD exhibit stronger visual participation than do those without when performing a series of perceptual or cognitive tasks (32, 33).

During cognition and verbal communication, individuals with ASD rely on their visual ability to acquire information through visual stimuli. The AVM method involves the application of visual support strategies and visual media as training. Out of the target behaviors, many international studies have confirmed that the video model law is consistent in children with ASD. In the present study, we found that the use of a tablet computer to play videos can better stimulate and maintain patients’ viewing motivation and observation ability and enable students to acquire and maintain the target behavior further by watching visual cues.

While fNIRS has emerged as a promising tool in neurocinematics, demonstrating significant inter-subject correlations in brain responses during multimedia experiences (34), other neuroimaging techniques such as functional Magnetic Resonance Imaging (fMRI) and Electroencephalography (EEG) have been more commonly applied in different contexts, including cognitive load assessment in multimedia learning (35) and driving behavior research (36). Despite the advantages of fNIRS, such as portability and reduced sensitivity to movement artifacts (37), the overall application of diverse neuroimaging methods in AVM remains underexplored. This gap highlights the need for further research to leverage various neuroimaging techniques to enhance understanding of cognitive and emotional responses elicited by animated video content.

Furthermore, children with ASD reportedly exhibit atypical visual perception abilities and a decrease in functional connectivity between the OL and IPL (38–40). Additionally, compared with that in a quiet state, functional connectivity between the OL and IPL was enhanced in children with ASD when they watched demonstration videos. IPL is linked to the ventral premotor cortex and plays a crucial role in processing visual and somatosensory information (41); this could be attributed to the rapid attention-grabbing nature of the demonstration videos on electronic screens for children with ASD, serving to stimulate their visual cortical responses. Conversely, in TD children, functional connectivity between the DLPFC and other brain regions, as well as between the mPFC and other brain regions, was lower when they watched demonstration videos than when they were in a quiet state. This may be due to differences in the behavior and cognitive mechanisms of TD children when watching videos, which may be influenced by their knowledge and experience. It has also been hypothesized that audiovisual modulation enhances the visual perception and information processing abilities of children with ASD by increasing the functional connectivity strength between the OL and IPL, thus serving as a potential treatment approach for ASD.

The use of virtual reality (VR) to enhance the social and cognitive skills of children with ASD has garnered widespread attention and interest across various sectors (42–44). The key feature of VR technology is its ability to provide a controlled visual and auditory experience (45, 46), allowing for the real-time assessment of patient adaptation and acceptance levels within the virtual training environment, thereby enabling appropriate adjustments. Integrating VR technology into social interaction demonstration videos offers novel methods and concepts for developing personalized rehabilitation programs for individuals with ASD.

The literature on visual tasks applied to the ASD population reveals a significant gap in recent studies, particularly regarding the integration of visual perception skills and their implications for interventions. A systematic review identified only 16 relevant studies on visual perception in ASD from 2014 to 2024, highlighting diverse findings such as enhanced visual processing and perceptual biases, yet underscoring the need for tailored interventions to address visual perception deficits that impact daily functioning (47). Additionally, a review of computer vision applications from 2009 to 2019 indicated that while computer vision techniques have potential in quantifying behavioral markers in ASD, the overall impact on visual task research remains underexplored (48). Furthermore, the application of virtual reality for vocational skills training in ASD has shown promise, but calls for more research to ensure skill maintenance and generalization (49). Overall, the current state of literature suggests a pressing need for updated studies focusing on visual tasks in ASD to better understand and leverage these unique perceptual characteristics (50, 51).

However, fNIRS presents several limitations, particularly regarding its penetration depth and spatial resolution compared to other neuroimaging techniques like functional magnetic resonance imaging (fMRI). While fNIRS is advantageous for its portability and ability to tolerate motion, it primarily measures hemodynamic responses in superficial cortical regions, which restricts its effectiveness in studying deeper brain structures (52). The technique’s reliance on scalp-located sensors and the diffusive nature of light in biological tissues complicate accurate hemoglobin evaluation, leading to potential errors in data interpretation due to hemoglobin cross talk (53). Additionally, variations in cap placement and systemic noise can further compromise signal quality, making it challenging to achieve reliable and repeatable measurements (54). Consequently, while fNIRS is a valuable tool for certain applications, it requires careful consideration, especially in studies requiring comprehensive functional connectivity assessments.

### Study limitations

This study had limitations. First, due to constraints of the detecting equipment, the brain functional connectivity data included only the frontal, parietal, and occipital lobes, excluding the temporal lobe. Second, investigation into screen time in children with ASD using demonstration videos was lacking; hence, future research should focus on screen viewing time and personalized video content customization. Third, owing to the limited number of participants, larger cohort studies are required to better understand the functional connectivity characteristics of the brains of children with ASD, as well as to provide new insights for evaluating and treating sensory perception in patients with ASD.

Although our study is premature to conclude definitively that AVM constitutes an “efficient” or standalone treatment for ASD, these results serve as an initial indication of AVM’s potential to support visual processing in ASD, justifying the need for further investigation with larger cohorts, more robust experimental controls, and long-term follow-up assessments.

## Data Availability

The raw data supporting the conclusions of this article will be made available by the authors, without undue reservation.
